# Impact of the Stem Borer, *Dectes texanus*, on Yield of the Cultivated Sunflower, *Helianthus annuus*.

**DOI:** 10.1673/031.007.2101

**Published:** 2007-04-12

**Authors:** J.P. Michaud, Angela K. Grant, J.L. Jyoti

**Affiliations:** ^1^ Kansas State University, Agricultural Research Center - Hays, 1232 240^th^ Ave, Hays, KS, 67601, 785-625-3425

**Keywords:** *Ataxia hubbardi*, *Cylindrocopturus adspersus*, girdling, insects, lodging, *Mordellistena* sp., oil content, *Pelochrista womanana*, seed weight, yield

## Abstract

Foliar and soil-drench insecticide treatments were used in attempts to manipulate infestation of cultivated sunflower plants, *Helianthus annuus* LeConte (Asterales: Asteraceae) by *Dectes texanus* LeConte, (Coleoptera: Cerambycidae) a serious pest of sunflowers in the High Plains of the USA. Seed yields were assessed on a per-plant basis for both oilseed and confection type sunflower hybrids in two years. Both insecticide treatments (foliar ë-cyhalothrin and soil-drench carbofuran) improved yield of oilseed sunflowers in 2004, but not in 2005. Yield of confection hybrids was improved by a systemic fungicide (thiophanate methyl) in 2005, but insecticides did not improve yield in either year. Both insecticide treatments gave good control of various stalk-boring insects such as *Cylindrocopturus adspersus* (Coleoptera: Curculionidae), *Mordellistena* sp. (Coleoptera: Mordellidae), and *Pelochrista womanana* (Lepidoptera: Tortricidae), but neither gave better than 50% control of *D. texanus*. Plants were sorted according to the presence or absence of *D. texanus* larvae and no reduction was found in total seed weight, seed size, or oil content as a result of infestation. However, mature larvae of D. *texanus* girdle stalks at the base in preparation for overwintering, a behavior that reduced stalk breakage force by 34–40%, leading to yield losses through lodging. At harvest in 2005, there were differences between cultivars and among treatments in the proportions of *D. texanus* larvae that had girdled their plants at harvest. It was concluded that further research aimed at reducing crop losses to *D. texanus* should focus on means of delaying stalk desiccation and/or deterioration, factors that appear to trigger girdling behavior.

## Introduction

The indigenous longhorned beetle, *Dectes texanus* LeConte, has become a serious pest of cultivated sunflowers (Philips et al. 1973; Rogers 1985) and soybeans (Falter 1969; Hatchett et al. 1975; Campbell and van Duyn 1977) in the American midwest. Originally described from the wild sunflower *Helianthus annuus* (LeConte 1862), the natural host plants of *D. texanus* are weeds such as *Ambrosia* and *Xanthium* spp. (Compositae) (Hatchett et al. 1975), making its pest status in soybeans somewhat anomalous. Yield losses in both crops arise when stalks of mature plants are girdled from the interior by larvae in preparation for overwintering, causing plants to lodge either prior to, or during, harvest.

There is only one generation per year, and late-instar larvae of *D. texanus* overwinter in the stubble of their host plants. Adult beetles emerge over an extended period from late spring to mid-summer and females begin to lay eggs at about two weeks of age (Crook et al. 2004; Michaud and Grant 2005). Little is known of the range of adult dispersal, but insects likely move only as far as necessary to find suitable food plants (Michaud and Grant 2005). Female feeding behavior is strongly correlated with egg laying (Michaud and Grant 2005) and oviposition punctures are normally initiated in feeding scars (Hatchett et al. 1975). Eggs are laid in the leaf petioles (Hatchett et al. 1975) and hatching larvae bore down the petiole into the core of the main stalk where they feed on the pith. In soybean, this behavior leads to premature leaf senescence and infested plants can be identified early in the season by the presence of wilted leaves. There is no tendency for females to avoid previously attacked plants, and multiple eggs are often laid by a single female in the same plant, or even the same petiole (Hatchett et al. 1975; Michaud and Grant 2005). Larvae are highly aggressive toward conspecifics and eliminate one another by combat until typically only one larva remains in a plant by season's end. As the stalk dries down, the larva moves to the base of the plant, prepares an overwintering chamber, and then girdles the base of the stalk above the chamber before plugging the stalk cavity with shredded fibers.

Michaud and Grant (2005) showed that *D. texanus* females preferred cultivated sunflower to soybean for both feeding and oviposition and that soybean appears to be a relatively inferior host plant for both larvae and adults, leading to significantly reduced pupal weight and shorter adult life. Since cultivated sunflower fields in the High Plains frequently have 80% or more of plants infested by *D. texanus*, it was of interest to know whether or not the boring damage done by larvae had an impact on the productivity and yield of sunflower plants independent of the losses attributable to lodging of girdled plants. In sunflowers grown for the confection market, premiums are paid for large seed size, whereas in the oilseed market, premiums are paid for high oil content. The present study was initiated with the objective of measuring sunflower seed yields on a per-plant basis to determine whether or not *D. texanus* larval boring affected plant productivity in terms of either total seed weight, seed size, or oil content. Various insecticide treatment regimes were applied with the objective of generating a series of plants with and without *D. texanus* larvae and then protecting the yield of these plants from other sources of damage until harvest. However, other species of Coleoptera and Lepidoptera also cause boring damage to sunflower stalks in the area of this research, potentially confounding measurements of *D. texanus* yield impact. Primary among these are the sunflower stem weevil, *Cylindrocopturus adspersus* (LeConte) the sunflower root moth, *Pelochrista womanana* Kearfoot, and the tumbling flower beetle, *Mordellistena* spp. Fortunately, these species are all effectively controlled by insecticide treatments that are far less effective against *D. texanus*.

## Materials and Methods

Experiments were conducted at Kansas State University, Agricultural Research Center- Hays, KS (38° 51′N 99° 20′W) on a crete silty clay loam soil (22% sand, 48% silt, and 30% clay) with pH 6.2 and 1.896 organic matter. The confection hybrid used was Triumph 757c and the oilseed variety was a Nusun hybrid, Triumph 665. In each year of the study, a field of 0.6 hectares was planted ‘no-till’ with each hybrid variety using a John Deere plated planter at a 76 cm row spacing. All fields were fallow the year prior to planting sunflowers. Prior to planting, fields were fertilized with 57 kgs N per acre in the form of anhydrous ammonium and then given a pre-emergent herbicide treatment of 2.0 L / ha S-metolachlor (Dual II Magnum, Syngenta Crop Protection Inc., www.syngenta.com) and 0.225 L / ha sulfentrazone (Spartan DF, FMC Corp., www.fmc.com). Following seedling emergence, plants were hand-thinned to a density of approximately 45,000 stems / ha (confection) or 55,000 stems / ha (oilseed). In 2004, both fields were planted on 14 May and emergence was noted on 21 May. Seedling destruction by black-tailed jack rabbits, *Lepus californiens*, reduced plant population to ca. 30,000 stems / ha in the confection field. The stand in the oilseed field was so poor that it had to be replanted on 4 June. In 2005, a rabbit control program was initiated in April and seedling losses were minimal. In order to achieve similar planting dates for each cultivar for the two years, the confection hybrid was planted on May 11 and the oilseed hybrid on June 6. There was sufficient moisture for prompt germination in both cases. Weather data was obtained for both growing seasons from the KSU, ARCH weather station located within 100m of the experimental fields.

The experiment was laid out in a completely randomized design with five replications of each treatment. When plants reached the 4–6 leaf stage, a series of 15 test plots were staked out in each field. Each test plot consisted of a section of a row with 30 plants or, in some cases, two adjacent rows with 15 plants in each. Plots were randomly assigned to one of three treatments: Control (no insecticide applied); λ-cyhalothrin (Warrior with Zeon technology, Syngenta) applied twice as a foliar spray at the equivalent of 0.288 L / ha, once at the 6-leaf stage (V6) and again at the12-leaf stage (V12), and carbofuran (Furadan 4F, FMC) applied twice as a soil drench at the equivalent of 0.720 L / ha, once at V6 and again at V12. In 2005, an additional treatment was added in the confection field which consisted of three soil drenches of the fungicide thiophanate methyl (3336F, Cleary Chemical Corp. www.clearychemical.com) at a rate equivalent to 4.8 L / ha, once each at V6, V12 and flower bud initiation (R1).

At bloom, all flower heads in test plots were dusted with a *Bacillus thuringensis* formulation (Dipel Dust, Southern Agricultural Insecticides Inc. www.southernag.com) every three days to protect against seed damage by sunflower moth, *Homoeosoma ellectulum* Hulst while sparing hymenopterous pollinators. At this time, a series of 15 plants in each test plot were selected for continued protection from seed-damaging pests, henceforth referred to as ‘protected yield’ plants. Flagging tape was used to tag the head and stalk of each selected plant with a numerical code indicating treatment, plot, and plant number. At petal fall, each selected flower head was covered with a brown paper bag secured with staples to prevent seed loss to bird predation. Once plants reached physiological maturity, heads were harvested in their bags and dried in an oven at 38 °C for a period of 20–30 days. Harvest dates were 11 September and 23 September, 2004 and 2 September and 13 September, 2005, for confection and oil cultivars, respectively. The stalks of protected plants, including the roots, were then uprooted and brought to the laboratory where each stalk was measured for basal circumference at soil level and then carefully dissected to identify and tally all the stalk-boring insect larvae.

After completion of the drying process, flower heads were removed from bags, measured vertically and horizontally to estimate flower diameter, and then threshed individually using an improvised mechanical single-head threshing machine. The seed was cleaned, analyzed for moisture content using a Grain Analytical Computer (GAC 2000, Dickey John Corp. www.dickey-john.com), and then weighed on an analytical balance. A sample of 100 randomly-selected seeds was then counted out and weighed to provide an estimation of seed size. All seed weights were corrected for moisture content prior to analysis. Subsamples of 40 ml. were taken from each oilseed flower head and placed in a labeled envelope for subsequent determination of oil content by nuclear magnetic resonance (Gambhir and Agarwala 1985).

In 2005, measurements were made of stalk strength on the plants remaining in all test plots on the day after harvest of protected yield plants. This was accomplished with a digital force gauge (FGE-50X Dart, Shimpo Instruments, www.shimpoinst.com) attached to a 16 gauge wire looped around the stalk at a height of 50 cm. Force was applied to the wire loop until the stalk broke, whereupon the peak force was recorded and the circumference of the stalk measured basally at the point of breakage. Since stalk breakage force demonstrates a positive linear relationship with stalk circumference, force measurements were divided by basal circumference in order to generate a value of force per unit basal circumference (henceforth referred to as the ‘adjusted stalk breakage force’) so that stalk strength could be compared among treatments independently of variation in stalk girth. Since some plants had been girdled by *D. texanus* and others not, the presence / absence of girdling in the stalk was also noted. In the oilseed field, many girdled plants had lodged so the proportion of lodged plants was tallied in all plots. Data on rates of *D. texanus* infestation from the protected yield plants were used in each plot to estimate the proportions of *D. texanus* larvae that had girdled their plants prior to harvest.

**Figure 1.**
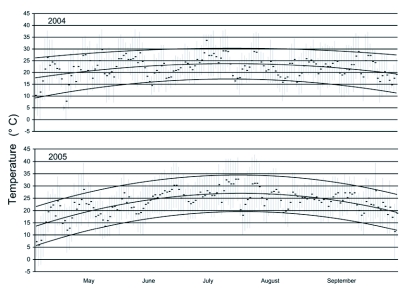
Maximum, minimum and median daily temperatures in experimental fields at the Agricultural Research Center - Hays during the growing seasons of 2004 and 2005. Curved lines depict seasonal trends.

Data for insects and components of yield were analyzed for effects of treatment separately for each hybrid using Proc. GLM (SAS Institute 1998) followed by Tukey's honestly significant different (HSD) test (α < 0.05) for separation of means. For each hybrid in each year, plants were then grouped according to presence/absence of *D. texanus* larvae and compared for components of yield using ‘treatment’ and ‘plot’ as fixed factors. Regression analyses were then used to test relationships between flower diameter, stalk circumference, and various components of yield. A χ^2^, goodness of fit test was used to compare rates of oilseed plant lodging in insecticide treatments to those in control plots in 2005.

## Results

### Weather

**Figure 2.**
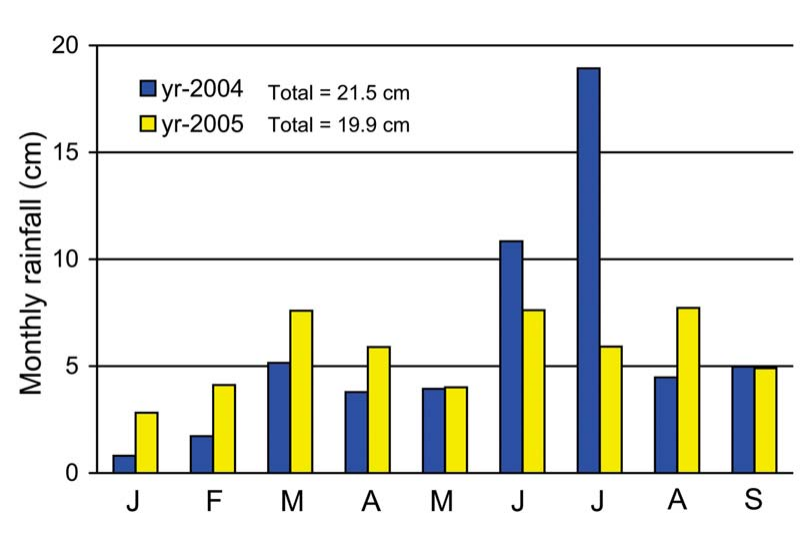
Monthly rainfall recorded in experimental fields at the Agricultural Research Center - Hays from January to September in 2004 and 2005.

The summer of 2004 was substantially cooler than that of 2005 (Figure 1). Although the two years had similar total rainfall by end of September, the growing season of 2004 began with much drier conditions in spring, but received more than twice the rainfall during June and July than in 2005 (Fig. 2), the two months during which sunflowers achieve most of their growth.

The confection field, established almost one month earlier than the oilseed field, was severely stressed by a strong wind storm on 4 July, 2004 that snapped off some plants and left many others lying prostrate on the ground. Surviving plants were provided special care (stalks and heads were supported off the soil) until maturity and most yielded well, but it was impossible to estimate the yield impact of this wind damage that occurred at a time when most plants were in full bloom. In contrast, the later-planted oilseed flowers were still in vegetative stages during the storm and were not adversely impacted beyond some leaf punctures caused by hailstones.

### Insect infestation

**Figure 3.**
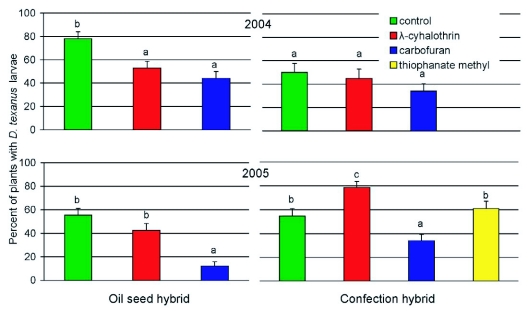
Effect of various pesticide treatments on mean percentages (± SEM) of oilseed and confection sunflowers infested by *Dectes texanus* larvae in two years at the Agricultural Research Center - Hays. Columns bearing the same letter were not significantly different (Tukey HSD, α < 0.05).

The rate of infestation of control oilseed plants by *D. texanus* was significantly higher in 2004 than in 2005 (*F* = 10.655; df = 1,146; *P* = 0.001) but there was no difference between years for confection plants (*F* = 0.197; df = 1,131; *P* = 0.666). Patterns of *D. texanus* infestation across pesticide treatments are depicted in Fig. 3. There were significant differences among treatments in rate of infestation in oilseed plants in both 2004 (*F* = 8.685; df = 2,215; *P* < 0.001) and 2005 (*F* = 18.530; df = 1,218; *P* < 0.001). However, treatment differences were not significant for confection plants in 2004 (*F* = 1.496; df = 2, 200; *P* = 0.227). The larger cerambycid, *Ataxia hubbardi* Fisher, which also bores the central pith core of stalks, infested 23.3, 15.5 and 16.5 percent of plants in the control, λ-cyhalothrin, and carbofuran treatments, respectively. This species was not observed in oilseed plants in either year, but is known to be more aggressive than *D. texanus* in larval combat (Michaud and Grant 2005). Although the treatments did not significantly affect infestation by *A. hubbardi* in 2004 (*F* = 0.625; df = 2,200; *P* = 0.536), it is likely that some larvae of *D. texanus* were eliminated from certain plants by this species earlier in the season.

**Table 1.**
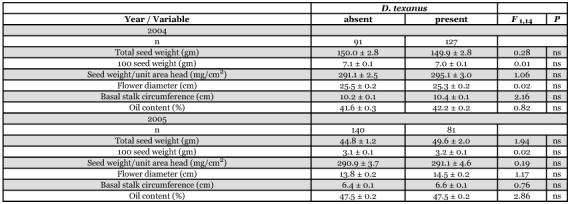
Mean (± SEM) values for components of yield for Triumph 665 oilseed sunflower plants with and without boring by larvae of *Dectes texanus*

There were significant effects of treatment on infestation by *D. texanus* in confection sunflowers in 2005 (*F* = 11.127; df = 3,292; *P* < 0.001). The percentage of plants with *D. texanus* larvae was similar in control and thiophanate methyl treatments, but the λ-cyhalothrin and carbofuran treatments significantly reduced *D. texanus* infestation (Figure 3). Treatment also affected infestation rate by *A. hubbardi* in 2005 (*F* = 13.504; df = 3,292; *P* < 0.001); control and thiophanate methyl treatments averaged more larvae per plant (0.22 ± 0.05 and 0.24 ± 0.05, respectively) than the λ-cyhalothrin (0.01 ± 0.01) or carbofuran (0.0) treatments.

**Table 2.**
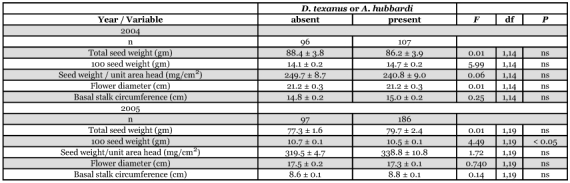
Mean (± SEM) values for components of yield for Triumph 757 confection sunflower plants with and without boring by larvae of *Dectes texanusor Ataxia hubbardi*.

Infestation of oilseed plants by other stem-boring insects was relatively low in both years of the experiment. In 2004, control plants contained an average of 5.5 ± 0.03 larvae of *C. adspersus*, 1.4 ± 0.01 larvae of *Mordellistena* spp., and 0.0 larvae of *P. womanana*. No larvae of these insects were found in plants receiving either of the insecticide treatments. In 2005, oilseed control plants contained an average of 0.76 ± 0.18 *C. adspersus* larvae, 0.12 ± 0.04 *Mordellistena* spp. larvae, and 0.05 ± 0.03 *P. Womanana* larvae, but no larvae of these species were found in plants receiving either of the insecticide treatments.

Infestation of confection plants by various stalk-boring insects was sufficient to permit statistical comparisons among treatments. In 2004, control plants contained a mean of 11.5 ± 1.02 *C. adspersus* larvae per plant, significantly more (*F* = 85.216; df = 2,200; *P* < 0.0001) than plants treated either with λ-cyhalothrin (2.1 ± 0.54) or carbofuran (1.04 ± 0.23). Control plants also contained more *Mordellistena* sp. larvae (0.65 ± 0.15) than either λ-cyhalothrin- or carbofuran-treated plants (0.08 ± 0.04 and 0.15 ± 0.05, respectively, *F* = 11.621; df = 2,200; *P* < 0.001). No larvae of *P*. *womanana* were recovered from plants in any treatment.

**Table 3a.**
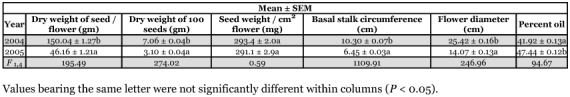
Effects of year on components of yield for oil seed sunflowers (Triumph 665) across all treatments.

In 2005 confection sunflowers, infestation by *C. adspersus* larvae averaged 19.1 ± 2.43, 6.0 ± 0.76, 0.03 ± 0.03, and 0.0 larvae per plant, respectively, in control, λ-cyhalothrin, carbofuran and thiophanate methyl plants (*F* = 50.981; 3,292; P < 0.0001). There was a significant positive relationship between numbers of *C. adspersus* larvae and total seed weight of control plants (*F* = 8.48; df = 70; *P* = 0.005; r2 = 0.108) and the basal circumference of these plants (*F* = 39.82; df = 70; *P* < 0.001; r2 = 0.363). Infestation by *Mordellistena* sp. averaged 0.86 ± 0.13, 0.0, 0.03 ± 0.02 and 1.0 ± 0.13 larvae per plant in the four treatments, respectively (*F* = 35.209; df = 3,292; *P* < 0.0001). Control confection plants contained a mean of 0.62 ± 0.08 *P. womanana* larvae per plant, significantly more (*F* = 37.481; df = 3,292; P < 0.0001) than any pesticide treatment, the latter being not significantly different from one another with 0.04 ± 0.02, 0.03 ± 0.02, and 0.11 ± 0.04 larvae per plant for the λ-cyhalothrin, carbofuran, and thiophanate methyl treatments, respectively.

**Table 3b.**
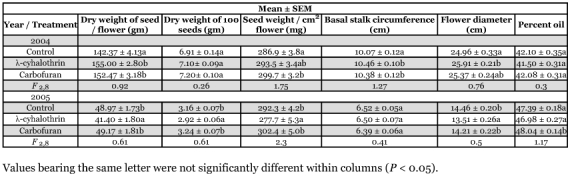
Effects of treatments on components of yield for oil seed sunflowers (Triumph 665) within years.

### Impact of *Dectes texanus* on components of yield

Although chemical treatments reduced infestation by *D. texanus*, they did not generate a level of differential infestation sufficient to permit analysis of yield impact by plot or treatment. Consequently, plants were sorted into two groups according to presence/absence of *D. texanus* larvae, ignoring treatment, using plot as a fixed factor. Analysis of data for the oilseed hybrid revealed that plants with and without *D. texanus* larvae were not significantly different with respect to any component of yield in either 2004 or 2005 (Table 1). For confection sunflowers, plants infested by *A. hubbardi* were included together with those infested by *D. texanus* since larvae of both insects cause similar plant damage by hollowing out the pith core of the stalk during the growing season. Once again, there was no significant effect of cerambycid infestation on any component of yield in either year (Table 2), except that the mean weight of 100 seeds from plants containing larvae was significantly lower than that from uninfested plants in 2005, despite a non-significant trend in the opposite direction in 2004.

### Components of yield for oilseed sunflowers

All components of oilseed yield were higher on a per-plant basis in 2004 than in 2005, except for seed weight per unit area of flower that did not differ, and oil content that was significantly lower (Table 3a and Table 3b). In 2004, both insecticide treatments resulted in increased stalk size, measured as basal circumference, and increased yield, measured as dry weight of seed per flower, without affecting seed size, as estimated by weight of 100 seeds, or oil content. This yield increase was associated with an increase in flower size in the λ-cyhalothrin treatment, but not in the carbofuran treatment, where it was associated with an increase in flower productivity (seed weight per unit area of head). There were no significant effects of treatment on seed oil content in 2004. In 2005, the λ-cyhalothrin treatment appeared to reduce components of yield compared to the carbofuran and control treatments. This yield reduction was accompanied by reductions in flower size, weight of 100 seeds, and productivity per unit area of flower head. In 2005, the carbofuran treatment improved seed oil content over that observed in either control or λ-cyhalothrin-treated flowers that did not differ from each other.

**Figure 4:**
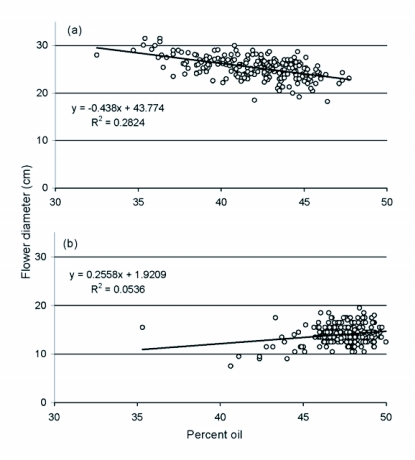
Linear regressions of seed oil content on flower diameter in 2004 (a) and 2005 (b) for Triumph 665 sunflowers grown at the Agricultural Research Center - Hays.

Linear regression revealed that seed oil content was negatively correlated with flower diameter in 2004 (*F* = 85.02. df = 216; *P* < 0.0001; r^2^ = 0.282; Figure 4a), and that seed size, as estimated by weight of 100 seeds, exhibited a positive relationship with flower diameter (*F* = 66.56; df = 216; P < 0.0001; r^2^ = 0.236). By contrast, in 2005, oil content exhibited a slightly positive relationship with flower diameter (*F* = 12.45; df = 220; *P* = 0.001; r^2^ = 0.054; Figure 4b) and the relationship between flower diameter and seed size was not significant (*F* = 2.45; df = 220; *P* = 0.119). However, the relationship between flower diameter and stalk circumference was strongly positive in both years (2004: *F* = 72.43; df = 216; *P* < 0.0001; r^2^ = 0.251; 2005: *F* = 48.78; df = 219; *P* < 0.0001; r^2^ = 0.182).

### Components of yield for confection sunflowers

All components of yield were higher on a per plant basis for confection flowers in 2004 than in 2005 (Table 4a and Table 4b). The smaller flowers in 2005 resulted in a greater weight of seed per unit area of head, although the seeds themselves were substantially smaller. Neither insecticide treatment affected any component of yield significantly in 2004, but sample sizes were reduced by wind damage in that year. In 2005, the thiophanate methyl treatment produced more weight of seed per flower than either the control or carbofuran treatments, apparently as a result of greater flower productivity measured as seed weight per unit area of flower. The λ-cyhalothrin treatment was not significantly different from any other treatment. However, the control and λ-cyhalothrin treatments resulted in larger seeds than either the carbofuran or thiophanate methyl treatments.

**Table 4a.**
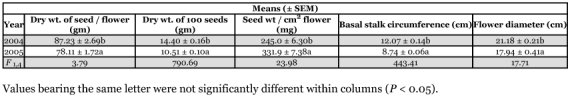
Effects of year on components of yield for confection sunflowers (Triumph 757c) across all treatments.

The flower diameter of confection plants was strongly and positively correlated with seed size (weight of 100 seeds) in both 2004 (*F* = 62.92; df = 201; *P* < 0.0001; r^2^ = 0.238) and 2005 (*F* = 65.50; df = 293; *P* < 0.0001; r^2^ = 0.188). The relationship between flower diameter and stalk circumference was also strongly positive in both years (2004: *F* = 81.27; df = 201; *P* < 0.0001; r^2^ = 0.288; 2005: *F* = 133.54; df = 288; *P* < 0.0001; r^2^ = 0.317).

### Girdling, lodging, and stalk breakage forces

**Figure 5.**
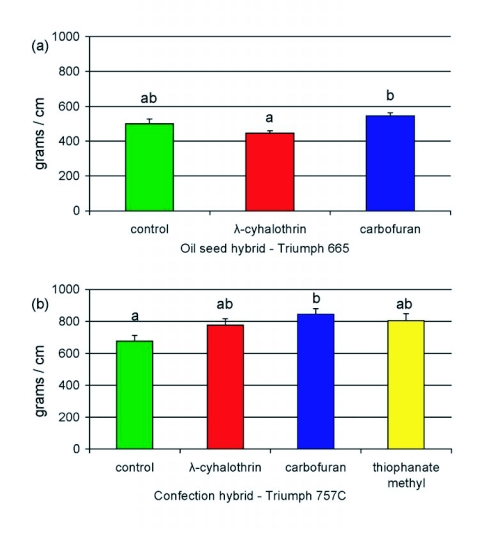
Mean (± SEM) adjusted stalk breakage forces (grams of force per unit cm basal stalk circumference) measured at harvest for two sunflower hybrids receiving various pesticide treatments in 2005 at the Agricultural Research Center - Hays. Columns bearing the same letter were not significantly different (Tukey HSD, α < 0.05).

Girdled oilseed stalks had a mean adjusted breaking force of 330 ± 28.6 gm/cm circumference compared to a mean of 502 ± 11.0 gm/cm for non-girdled stalks, corresponding to a 34.3% reduction in stalk strength. Similarly, girdled confection stalks had a mean adjusted stalk breakage force of 463.6 ± 174.0 gm/cmcircumference compared to a mean of 774.5 ± 20.5 gm/cm for ungirdled stalks, corresponding to a reduction of 40.1%. Considering only ungirdled plants, the carbofuran treatment significantly increased the adjusted stalk breakage forces for both oilseed plants (*F* = 8.551; df = 2,8; α < 0.05; Figure 5a) and confection plants (*F* = 3.361; df = 3,11; α < 0.10; Figure 5b).

**Table 4b.**
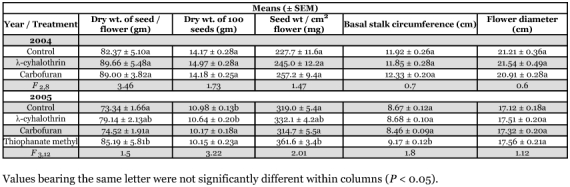
Effects of treatments on components of yield for confection sunflowers (Triumph 757c) within years.

In the oilseed field, 36.9% of control plants had lodged and were lying on the ground by date of harvest as a result of *D. texanus* girdling. This was significantly greater than the 7.9% lodging observed in λ-cyhalothrin-treated plants (χ2 = 44.33, *P* < 0.02) and the 0.6% lodging observed in carbofuran-treated plants (χ2 = 10,472.11, *P* < 0.001). The mean rates of *D. texanus* infestation obtained from protected yield plants in these treatments were used to estimate that 66.6% of larvae completed the girdling behavior in control plots, compared to 18.6% in λ-cyhalothrin plots, and 5% in carbofuran plots. The frequency of girdling in non-lodged plants that was used for stalk breakage force measurements was determined and were significantly reduced for the insecticide treatments (*F* = 23.489; df = 2,8; *P* < 0.0001), 33.3% for the control, versus 6.4% and 0.0% for the λ-cyhalothrin and carbofuran plots, respectively, were girdled in the three treatments (Tukey HSD, *P* < 0.05).

Very few plants had lodged as a result of girdling in the confection field by date of harvest (5 plants in control plots, 0 plants in λ-cyhalothrin and carbofuran plots, and 8 plants in thiophanate-methyl plots). Examination of plants used for stalk breakage measurements revealed that 14.0%, 13.2%, 0.0%, and 7.5% of plants were girdled by *D. texanus* in control, ë-cyhalothrin, carbofuran and thiophanate methyl plots, respectively, but these differences were not statistically significant (*F* = 2.367; df = 3,11; α > 0.05).

## Discussion

### Efficacy of treatments

Although both insecticide treatments appeared effective in controlling infestation by sunflower stem weevil, tumbling flower beetle, and root moth, neither could be considered adequate for control of *D. texanus*. *λ*-cyhalothrin has been shown to have high toxicity to *D. texanus* in the laboratory (Kazmareck et al. 2004) but adult beetles are active in the field for an extended period in summer and may have laid eggs in plants during periods when residual activity of the material was low. Failure of application timing to correspond adequately with female *D. texanus* activity is the most likely explanation for the failure of λ-cyhalothrin to reduce rates of larval infestation in oilseed plants in 2005 and in confection plants in either year, although why this treatment appeared to increase infestation in confection plants in 2005 is difficult to surmise. Campbell and van Duyn (1977) tested various insecticides and application techniques against *D. texanus* in soybeans and concluded that their treatments were ineffective against larvae and that numerous, carefully timed applications would be necessary to control adults.

The carbofuran treatment targeted larvae within plants and was applied as a soil drench to obtain good systemic activity. It may be that failure of this material to provide adequate control of *D. texanus* is partially derived from the larva's propensity to confine its feeding to the central pith core of the plant, avoiding conductive tissues that would presumably contain most of the insecticide. Rogers (1975) noted that systemic insecticides were ineffective in controlling *D. texanus* larvae in either soybean or sunflower. The two-fold reduction in stalk circumference in oilseed plants in 2005 relative to 2004 may have brought more larvae into contact with conductive elements of the stalk and resulted in better control in the former year. Some of the differences observed in insecticide efficacy between the two years may also have resulted from differences in environmental conditions. The higher summer rainfall of 2004 compared to 2005 would be expected to result in more rapid weathering of λ-cyhalothrin residues on foliage, and perhaps improve plant uptake of the carbofuran soil drenches. However, such effects do not appear implicated in levels of *D. texanus* control because λ-cyhalothrin was generally less effective in 2005, whereas the reverse was true for carbofuran.

### Impact of *D. texanus* on sunflower yields

When treatments were pooled and all plants with cerambycid larvae were compared to those without, there was no effect of *D. texanus* larval boring on any component of yield (Tables 1 and 2) except in confection plants in 2005 when plants with larvae had reduced seed weights compared to those uninfested. However, the magnitude of the difference was small (0.2 gm) and the opposite trend, although non-significant, was evident in 2004, suggesting variation due to chance effects.

There are various possible explanations for why rate of *D. texanus* infestation was lower in oilseed plants in 2005 than in 2004 while this was not the case in confection plants. If the peak period of female oviposition occurred later in 2004, females may have oviposited more eggs in the later-planted oilseed cultivar relative to the confection cultivar in response to their earlier stage of development. However, it is also possible that the small stalks of oilseed plants in 2005 reduced their acceptabilty for oviposition by *D. texanus* females.

### Components of yield

Both insecticide treatments increased the seed yield of oilseed plants in 2004, although neither did so in 2005. Interestingly, the mechanisms of yield increase in 2004 appeared slightly different for the two materials tested; the λ-cyhalothrin treatment resulted in larger flowers than control plants, whereas the carbofuran treatment resulted in flowers that were more productive per unit area of head (Table 3). Both insecticide treatments increased stalk girth. Given the absence of stalk-feeding insects in control plants (apart from *D. texanus*), it seems likely that insects feeding on other plant parts were implicated in the observed yield losses. Although the foliar λ-cyhalothrin treatment would presumably be limited to impacts on foliage-feeding insects, the carbofuran soil drenches would presumably affect root, stem, and foliage insects to some degree. Thus defoliation might primarily impact flower size, whereas root and stem feeding might have more impact on flower productivity in terms of seed fill.

Although no particular folivorous insect was observed in numbers approaching an economic injury level in 2004, a complex of larval Lepidoptera were observed defoliating plants and may have collectively been responsible for reducing the mean stalk circumference, flower diameter, and seed weight of control oilseed plants. The primary species observed included *Estigmene acrea* (Drury) (Lepidoptera, Arctiidae) and *Vanessa cardui* L. (Lepidoptera: Nymphalidae). Muma et al. (1950) found variable effects of foliar insecticide treatment on sunflower yields in Nebraska that they attributed to differential impacts on lepidopterous larvae and their natural enemies in different years. However, only the λ-cyhalothrin treatment would have impacted beneficial insects active on sunflower foliage as the carbofuran was applied as a soil drench.

It is notable that the seed weight of oilseed sunflowers was 222% higher in 2004 relative to 2005, whereas it was only 11% higher for confection sunflowers in 2004 which was a superior growing season. However, it is difficult to understand this contrast because of the wind damage to confection plants during seed maturation in 2004 and because variability in stand establishment between the two hybrids in both years probably had significant effects on plant size. Since the thiophanate methyl treatment produced the highest seed yield in confection flowers in 2005, it is possible that stalk- and root-infecting fungi were more limiting to yield than were insects in that particular field and year. However, the effectiveness of both insecticide treatments in increasing the yield of oilseed plants in 2004 suggests that herbivorous insects (other than *D. texanus*) were limiting to yield in that particular year. The lack of such treatment effects in 2005 is consistent with factors other than herbivory, most likely soil moisture, being more limiting to yield. The reduced yields of λ-cyhalothrin-treated oilseed plants relative to controls in 2005 is difficult to explain, but may reflect a chance effect of these treatment plots being located in less productive parts of the field. Some bract necrosis induced by heat scorch was observed on oilseed flowers in 2005 and this damage impeded the development of some flowers, particularly in two λ-cyhalothrin plots.

Reliable yield data for confection plants was obtained only in 2005 and the λ-cyhalothrin treatment produced the best combination of seed weight and seed size, again suggesting the potential benefits of protecting foliage. The positive correlation between numbers of *C. adspersus* larvae and total weight of seed from control confection plants probably reflects the tendency of bigger stalks to harbor larger numbers of larvae, and clearly indicates that a mean of 19 larvae per stalk is well below an economic impact threshold for this pest. The consistent positive relationship between basal stalk circumference and flower diameter suggests that wider plant spacing is one tactic for increasing stalk girth along with flower size for confection varieties where seed size is also correlated with flower diameter. However, a negative relationship between flower diameter and seed oil content was evident in 2004 when oilseed flowers ranged between 20 and 30 cm in diameter, consistent with the inference that such large flowers are not desirable for oil production. Mean seed oil content was 13.4% higher in 2005 when most flowers ranged between 10 and 20 cm in diameter and the negative correlation with flower diameter disappeared, suggesting that this relationship might only be a concern when flowers become larger than 20 cm.

### Stalk breakage forces, lodging, and girdling behavior

Fungicide treatment increased the breakage forces of ungirdled stalks significantly more than either insecticide treatment in the 2005 confection plants, suggesting that fungi may play a role in stalk deterioration at crop maturity that is worthy of further investigation. Similarly, the generally higher stalk breakage forces obtained from the carbofuran treatments compared to the λ-cyhalothrin treatments are likely a function of the systemic activity of the former material in delaying various processes associated with stalk degradation, but data from multiple years would be required to confirm this.

Girdling by *D. texanus* larvae reduced stalk breakage forces by 34–40%. However, larvae appear physically unable to fully girdle sunflower stalks that are greater than 2.5 cm in diameter. Similarly, Wapshere (1974) noted that larvae of the cerambycid *Mecas saturnina* LeConte were only able to partially girdle cocklebur plants with stems > 2.0 cm in diameter. Confection sunflowers are planted at lower densities than oilseed plants with the goal of obtaining larger heads that, as our data clearly show, are not only associated with larger seeds, the criterion for premiums in confection markets, but also with thicker stalks. Farmers specializing in the production of confection sunflowers usually manage to avoid lodging problems simply because stout confection stalks are robust enough to remain standing even when girdled by a *D. texanus* larva. Note also that the stalk breakage forces tended to be 50% higher for the confection hybrid than for the oilseed hybrid after adjustment for differences in plant size, suggesting that the former were intrinsically stronger. Lodging losses caused by *D. texanus* are, therefore, a more serious concern for farmers growing oilseed cultivars, especially when they depend upon rain, as moisture is often limiting. Oilseed sunflowers grown at high density under dry conditions not only have slender stalks that can be completely girdled by *D. texanus*, but their stalks also dry out quickly after crop maturity, likely triggering earlier onset of the girdling behavior (see below). Reduction of planting density to increase stalk girth is one possible tactic for oilseed farmers, provided flower diameter head does not increase above 20 cm.

In 2005, a large proportion of untreated plants in the oilseed field had lodged by time of harvest, whereas this was not the case in untreated confection plants despite similar rates of *D. texanus* infestation. Although the larger girth of confection stalks may be implicated here, it was also apparent that fewer larvae had completed girdling in the confection hybrid than in the oilseed hybrid by date of harvest. There were also marked differences among treatments in completion of stalk girdling by larvae in the oilseed plants in 2005, as both insecticide treatments appearing to delay the behavior. One explanation consistent with these observations is that girdling behavior may be triggered by stalk desiccation because this determines the end of its suitability as a food source. We have often reared *D. texanus* larvae on fresh sunflower stalks in the laboratory, but have never managed to elicit feeding on dry stalk material. In contrast, larvae of *A. hubbardi* do not girdle plants and readily feed and gain weight when provided with completely dry stalk pith (AKG, personal observation). The absence of lodging in both cultivars in 2004 is consistent with the moist conditions that prevailed up until harvest in that year. The oilseed stalks in 2005 suffered substantial lodging, but were considerably more slender than confection stalks, and also notably more dry and brittle at time of harvest. Both large plant size and pesticide treatment might delay stalk desiccation and this effect could explain the observed delays in girdling behavior in confection plants relative to oilseed plants and in pesticide treatments relative to controls. Similarly, Richardson (1975) concluded that desiccation of pith within stems represented the end of a suitable food supply and was a likely trigger for *D. texanus* girdling behavior in soybeans. Anecdotal support for this interpretation is provided by observations of commercial fields where non-irrigated regions and dry spots typically exhibit lodging much earlier than moist or irrigated portions, despite similar rates of larval infestation.

Given that larval boring by *D. texanus* larvae has no measurable impact on yield, further research directed at mitigating losses to this pest should focus on factors triggering the girdling behavior and possible means of delaying or otherwise manipulating this behavior. Possibilities for further investigation include systemic materials that delay stalk deterioration, dessicants that accelerate drying of flower heads relative to stalks, or the development of cultivars with ‘stay-green’ stalks. The common objective of these approaches would be to delay the onset of girdling behavior by *D. texanus* larvae until well after seed has dried down to harvestable levels.
